# Bumetanide in Patients with Alzheimer's Disease (BumxAD): A Phase IIa Clinical Trial

**DOI:** 10.1002/alz70859_106859

**Published:** 2025-12-25

**Authors:** Mina Kmiecik, Minhtrang Chu, Annie Zhou, Irina Anna Skylar‐Scott, Niyatee Samudra, Pragya Tripathi, Maria Coburn, Ted N. Wilson, Zihuai He, Francois Haddad, Tony Wyss‐Coray, Sharon J Sha, Michael D. Greicius, Kyan Younes

**Affiliations:** ^1^ Stanford University, PALO ALTO, CA USA; ^2^ Stanford University, Palo Alto, CA USA; ^3^ Department of Neurology and Neurological Sciences, Stanford University School of Medicine, Stanford, CA USA; ^4^ Wu Tsai Neurosciences Institute, Stanford University, Stanford, CA USA; ^5^ Department of Neurology and Neurological Sciences, Stanford University, Stanford, CA USA; ^6^ Stanford University, Stanford, CA USA

## Abstract

**Background:**

Bumetanide is a potent diuretic administered orally and is FDA approved for the treatment of edema and hypertension. Recently, repurposing Bumetanide as an Alzheimer`s disease (AD) medication was proposed based on data that showed Bumetanide “flipped” the APOE genotype‐dependent transcriptomic signatures in AD mouse and cell culture models.^1^ Critically, this finding was then investigated in Electronic Health Record (EHR) cohorts and showed that in individuals over 65 years of age, bumetanide exposure was associated with a significantly lower AD prevalence in three independent dataset. However randomized double blinded trials are needed to investigate the safety, tolerability and benefit of bumetanide in AD.

**Method:**

This is a phase II, randomized, double‐blind, multi‐arm placebo‐controlled, parallel group study to evaluate the safety and tolerability of bumetanide in patients with Alzheimer’s disease. This study aims to investigate bumetanide in patients with biologically confirmed AD. The primary objective is to evaluate the safety and tolerability of bumetanide when administered to participants with biomarker‐confirmed AD. The secondary objective is to evaluate the clinical and biomarker effects of bumetanide in participants with mild cognitive impairment or mild dementia due to Alzheimer's disease.

**Result:**

This ongoing study is funded by the Stanford Knight Initiative for Brain Resilience and IRB approved. The inclusion criteria include mild cognitive impairment or mild dementia due to AD, AD medications are planned to remain stable throughout, willingness and ability to complete all aspects of the study including assessments, neuropsychological testing, and MRI. Exclusion criteria includes clinically significant abnormalities in screening laboratory tests, chronic liver disease, renal insufficiency, poorly managed hypertension and participants taking the following concomitant medications, based on the current Prescribing Information for bumetanide: lithium, drugs with ototoxic potential, drugs with nephrotoxic potential, probenecid, and indomethacin

**Conclusion:**

We present the clinical trial protocol and design for a phase II trial evaluating the effect of bumetanide in AD.

